# Genome Sequence of *Pseudomonas aeruginosa* Strain LCT-PA220, Which Was Selected after Space Flight by Using Biolog’s Powerful Carbon Source Utilization Technology

**DOI:** 10.1128/genomeA.00169-14

**Published:** 2014-03-13

**Authors:** Guogang Xu, Juan Hu, Xiangqun Fang, Xuelin Zhang, Junfeng Wang, Yinghua Guo, Tianzhi Li, Zhenghong Chen, Wenkui Dai, Changting Liu

**Affiliations:** a Nanlou Respiratory Diseases Department, Chinese PLA General Hospital, Beijing, China; b BGI-Shenzhen, Shenzhen, China

## Abstract

To explore the changes of *Pseudomonas aeruginosa* in space flight, we present the draft genome sequence of *P. aeruginosa* strain LCT-PA220, which originated from a *P. aeruginosa* strain, ATCC 27853, that traveled on the Shenzhou-VIII spacecraft.

## GENOME ANNOUNCEMENT

We describe the draft genome sequence of *Pseudomonas aeruginosa* strain LCT-PA220, which was selected using Biolog’s powerful carbon source utilization technology. The strain was derived from *P. aeruginosa* ATCC 27853, which was sent into space for 398 h on the Shenzhou-VIII spacecraft (1).

The genomic DNA of *P. aeruginosa* LCT-PA220 was used to construct short (350-bp) and long (6-kb) random sequencing libraries, with genome coverages of 100× and 50×, respectively. Whole-genome sequencing of 90-bp reads of both ends of the fragments was performed using an Illumina HiSeq 2000 system (Illumina, Inc., USA), according to the manufacturer’s instructions.

Using Short Oligonucleotide Analysis Package (SOAP)*denovo* version 1.05, we assembled the sequences into 194 contigs, with a total length of 6,746,593 bp, and subsequently into 45 scaffolds (≥500 bp in size), with 93,864-bp gaps. The *N*_50_ of the assembled scaffolds is 1,723,471 bp, and the G+C content is 66.17%. We used Glimmer version 3.02 to predict putative open reading frames on the scaffolds. From the analysis of the LCT-PA220 sequence, we found that the genome contains 6,464 genes and the total length of gene sequences is 6,089,727 bp, which comprises 88.78% of the genome. The coding sequences (CDSs) were annotated by alignment to the Swiss-Prot, COG, KEGG, TrEMBL, and NR databases.

Three rRNAs were predicted using RNAmmer, and 54 tRNA genes were predicted using tRNAscan-SE1.21. We also used RepeatMasker version 3.2.9 and RepeatProteinMasker to predict transposon sequences, and we used Tandem Repeats Finder (TRF) version 4.04 to predict tandem repeat sequences. We found that the number of tandem repeat sequences is 210, with a total length of 19,669 bp, which is 0.2868% of the entire genome sequence.

### Nucleotide sequence accession number.

This whole-genome sequence of *P. aeruginosa* LCT-PA220 has been deposited at DDBJ/EMBL/GenBank under the accession no. ATJT00000000. The version described in this paper is the first version.
